# Bis(2-amino­pyrimidin-1-ium) sulfate

**DOI:** 10.1107/S1600536812037725

**Published:** 2012-09-12

**Authors:** Hui-Ling Hu, Chun-Wei Yeh

**Affiliations:** a Department of Chemical and Material Engineering, Taoyuan Innovation Institute of Technology, Jhongli 32091, Taiwan; b Department of Chemistry, Chung-Yuan Christian University, Jhongli 32023, Taiwan

## Abstract

In the title compound, 2C_4_H_6_N_3_^+^·SO_4_^2−^, the cations are each essentially planar with r.m.s. deviations of the fitted atoms of 0.008 and 0.002 Å. In the crystal, adjacent ions are linked by N—H⋯O, C—H⋯O and C—H⋯N hydrogen bonds, forming a three-dimensional network.

## Related literature

For the crystal structures of 2-amino­pyrimidinium salts with other anions, see: Cheng *et al.* (2010 ▶); Eshtiagh-Hosseini *et al.* (2010 ▶). 
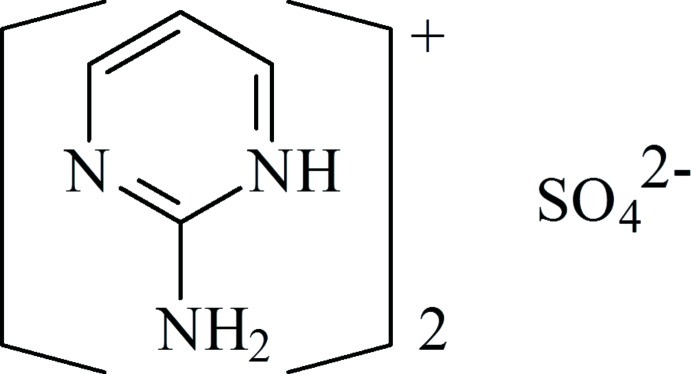


## Experimental

### 

#### Crystal data

2C_4_H_6_N_3_^+^·SO_4_^2−^
*M* *_r_* = 288.30
Monoclinic, 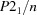

*a* = 8.1215 (8) Å
*b* = 11.4853 (12) Å
*c* = 13.0407 (14) Å
β = 97.206 (2)°
*V* = 1206.8 (2) Å^3^
*Z* = 4
Mo *K*α radiation
μ = 0.29 mm^−1^
*T* = 293 K
0.45 × 0.29 × 0.16 mm

#### Data collection

Bruker APEXII CCD area-detector diffractometer
Absorption correction: multi-scan (*SADABS*; Bruker, 2000 ▶) *T*_min_ = 0.880, *T*_max_ = 0.955
6656 measured reflections
2377 independent reflections
1976 reflections with *I* > 2σ(*I*)
*R*_int_ = 0.032

#### Refinement

*R*[*F* ^2^ > 2σ(*F* ^2^)] = 0.032
*wR*(*F* ^2^) = 0.093
*S* = 1.05
2377 reflections
196 parameters
H atoms treated by a mixture of independent and constrained refinement
Δρ_max_ = 0.19 e Å^−3^
Δρ_min_ = −0.40 e Å^−3^

### 

Data collection: *APEX2* (Bruker, 2010 ▶); cell refinement: *SAINT* (Bruker, 2010 ▶); data reduction: *SAINT*; program(s) used to solve structure: *SHELXS97* (Sheldrick, 2008 ▶); program(s) used to refine structure: *SHELXL97* (Sheldrick, 2008 ▶); molecular graphics: *DIAMOND* (Brandenburg, 2010 ▶); software used to prepare material for publication: *SHELXL97*.

## Supplementary Material

Crystal structure: contains datablock(s) I, global. DOI: 10.1107/S1600536812037725/pv2583sup1.cif

Structure factors: contains datablock(s) I. DOI: 10.1107/S1600536812037725/pv2583Isup2.hkl

Supplementary material file. DOI: 10.1107/S1600536812037725/pv2583Isup3.cml

Additional supplementary materials:  crystallographic information; 3D view; checkCIF report

## Figures and Tables

**Table 1:** Hydrogen-bond geometry (Å, °)

*D*—H⋯*A*	*D*—H	H⋯*A*	*D*⋯*A*	*D*—H⋯*A*
N1—H1*NA*⋯O1	0.86 (2)	1.99 (2)	2.853 (2)	175.5 (17)
N1—H1*NB*⋯O3^i^	0.91 (2)	1.97 (2)	2.882 (2)	176.9 (2)
N2—H2*N*⋯O2	0.86 (2)	1.79 (2)	2.640 (2)	174.7 (18)
N4—H4*NA*⋯O1	0.81 (2)	2.10 (2)	2.902 (2)	167.6 (19)
N4—H4*NB*⋯O3^ii^	0.78 (2)	2.19 (2)	2.962 (2)	171.0 (2)
N5—H5*N*⋯O4^ii^	0.80 (2)	1.84 (2)	2.631 (2)	172.6 (2)
C2—H2*A*⋯O4^iii^	0.93	2.50	3.295 (2)	144
C3—H3*A*⋯N6^iv^	0.93	2.58	3.382 (2)	145
C4—H4*A*⋯O1^v^	0.93	2.57	3.231 (2)	128
C7—H7*A*⋯O2^vi^	0.93	2.51	3.101 (2)	121
C8—H8*A*⋯O4^vii^	0.93	2.59	3.237 (2)	127

Symmetry codes: (i) 
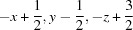
; (ii) 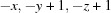; (iii) 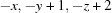; (iv) 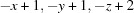; (v) 
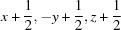
; (vi) 
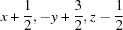
; (vii) 
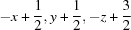
.

**Fig. 1.**
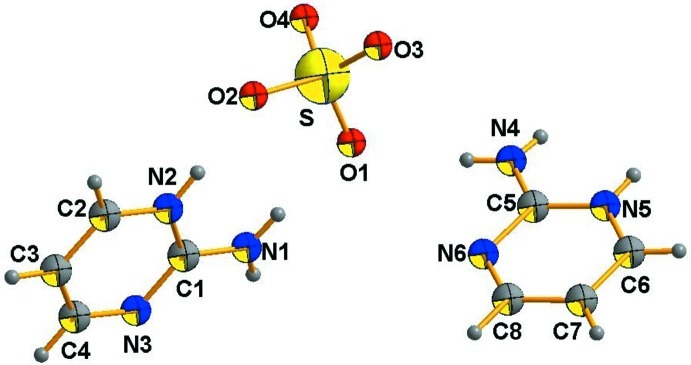
The molecular structure of the title compound with the atom numbering scheme. Displacement ellipsoids are drawn at the 30% probability level. H atoms are presented as small spheres of arbitrary radius.

**Fig. 2.**
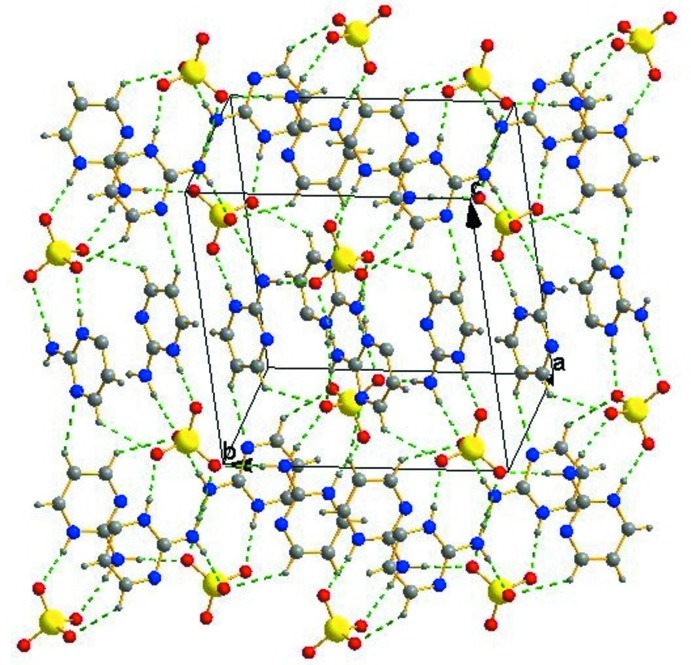
A view of the N—H···O, C—H···O and C—H···N hydrogen bonds (dotted lines) in the crystal structure of the title compound.

## supplementary crystallographic information

### Comment

There are several supramolecular structures containing 2-aminopyrimidinium cations with other anions constructed by hydrogen bonds (Cheng, *et al.* 2010; Eshtiagh-Hosseini, *et al.*, 2010). The asymmetric unit of the title compound, consists of two independent 2-aminopyrimidinium cations and a sulfate anion (Fig. 1). These two protonated pyrimidine rings are not co-planar but twisted with each other by an interplanar angle of 84.3 (1)°. The cations and anions are interlinked through N—H···O, C—H···O and C—H···N hydrogen bonds resulting in a three-dimensional net work (Fig. 2, Tab. 1).

### Experimental

An aqueous solution (5.0 ml) of zinc sulfate (1.0 mmol) was layered carefully over a methanolic solution (5.0 ml) of 2-aminopyrimidine (1.0 mmol) in a tube. Colourless crystals were obtained after several weeks. These were washed with methanol and collected in 85.8% yield.

### Refinement

H atoms bound to C atoms were placed in idealized positions and constrained to ride on their parent atoms, with C—H = 0.93 Å, and with *U*_iso_(H) = 1.2 *U*_eq_(C). The amine hydrogen atoms and the pyrimidinium hydrogen atoms were located in diffrernce Fourier maps and were allowed to refine with isotropic displacement parameters *U*_iso_.

### Crystal data

**Table:**

2C_4_H_6_N_3_^+^·SO_4_^2^^−^	*F*(000) = 600
*M**_r_* = 288.30	*D*_x_ = 1.587 Mg m^−^^3^
Monoclinic, *P*2_1_/*n*	Mo *K*α radiation, λ = 0.71073 Å
Hall symbol: -P 2yn	Cell parameters from 3511 reflections
*a* = 8.1215 (8) Å	θ = 2.5–26.0°
*b* = 11.4853 (12) Å	µ = 0.29 mm^−^^1^
*c* = 13.0407 (14) Å	*T* = 293 K
β = 97.206 (2)°	Plate, colourless
*V* = 1206.8 (2) Å^3^	0.45 × 0.29 × 0.16 mm
*Z* = 4	

### Data collection

**Table:**

Bruker APEXII CCD area-detector diffractometer	2377 independent reflections
Radiation source: fine-focus sealed tube	1976 reflections with *I* > 2σ(*I*)
Graphite monochromator	*R*_int_ = 0.032
φ & ω scans	θ_max_ = 26.0°, θ_min_ = 2.4°
Absorption correction: multi-scan (*SADABS*; Bruker, 2000)	*h* = −10→10
*T*_min_ = 0.880, *T*_max_ = 0.955	*k* = −14→8
6656 measured reflections	*l* = −16→14

### Refinement

**Table:**

Refinement on *F*^2^	Primary atom site location: structure-invariant direct methods
Least-squares matrix: full	Secondary atom site location: difference Fourier map
*R*[*F*^2^ > 2σ(*F*^2^)] = 0.032	Hydrogen site location: inferred from neighbouring sites
*wR*(*F*^2^) = 0.093	H atoms treated by a mixture of independent and constrained refinement
*S* = 1.05	*w* = 1/[σ^2^(*F*_o_^2^) + (0.0546*P*)^2^ + 0.1486*P*] where *P* = (*F*_o_^2^ + 2*F*_c_^2^)/3
2377 reflections	(Δ/σ)_max_ = 0.001
196 parameters	Δρ_max_ = 0.19 e Å^−^^3^
0 restraints	Δρ_min_ = −0.40 e Å^−^^3^

### Special details

**Table:**

Geometry. All e.s.d.'s (except the e.s.d. in the dihedral angle between two l.s. planes) are estimated using the full covariance matrix. The cell e.s.d.'s are taken into account individually in the estimation of e.s.d.'s in distances, angles and torsion angles; correlations between e.s.d.'s in cell parameters are only used when they are defined by crystal symmetry. An approximate (isotropic) treatment of cell e.s.d.'s is used for estimating e.s.d.'s involving l.s. planes.
Refinement. Refinement of *F*^2^ against ALL reflections. The weighted *R*-factor *wR* and goodness of fit *S* are based on *F*^2^, conventional *R*-factors *R* are based on *F*, with *F* set to zero for negative *F*^2^. The threshold expression of *F*^2^ > σ(*F*^2^) is used only for calculating *R*-factors(gt) *etc*. and is not relevant to the choice of reflections for refinement. *R*-factors based on *F*^2^ are statistically about twice as large as those based on *F*, and *R*- factors based on ALL data will be even larger.

### Fractional atomic coordinates and isotropic or equivalent isotropic displacement parameters (Å^2^)

**Table:**

	*x*	*y*	*z*	*U*_iso_*/*U*_eq_	
S	0.01967 (5)	0.48062 (3)	0.75579 (3)	0.03308 (15)	
C1	0.4028 (2)	0.33089 (14)	0.95900 (13)	0.0353 (4)	
C2	0.3254 (2)	0.46070 (16)	1.08388 (13)	0.0422 (4)	
H2A	0.2577	0.5207	1.1019	0.051*	
C3	0.4431 (2)	0.41520 (16)	1.15527 (15)	0.0498 (5)	
H3A	0.4602	0.4431	1.2227	0.060*	
C4	0.5372 (2)	0.32455 (17)	1.12244 (15)	0.0507 (5)	
H4A	0.6183	0.2919	1.1706	0.061*	
C5	0.32224 (19)	0.58057 (14)	0.50457 (12)	0.0328 (4)	
C6	0.4389 (2)	0.72057 (15)	0.40373 (14)	0.0435 (4)	
H6A	0.4403	0.7583	0.3408	0.052*	
C7	0.5485 (2)	0.75007 (17)	0.48542 (16)	0.0514 (5)	
H7A	0.6271	0.8081	0.4808	0.062*	
C8	0.5392 (2)	0.69007 (16)	0.57723 (15)	0.0483 (5)	
H8A	0.6150	0.7092	0.6342	0.058*	
N1	0.3820 (2)	0.29315 (15)	0.86293 (12)	0.0455 (4)	
H1NA	0.312 (2)	0.3295 (18)	0.8188 (16)	0.050 (6)*	
H1NB	0.435 (3)	0.227 (2)	0.8471 (16)	0.065 (6)*	
N2	0.30658 (17)	0.41906 (12)	0.98686 (11)	0.0362 (3)	
H2N	0.230 (2)	0.4489 (17)	0.9436 (15)	0.042 (5)*	
N3	0.51952 (18)	0.28162 (13)	1.02800 (12)	0.0458 (4)	
N4	0.2118 (2)	0.49912 (14)	0.51195 (14)	0.0425 (4)	
H4NA	0.207 (2)	0.4694 (18)	0.5680 (17)	0.047 (6)*	
H4NB	0.157 (2)	0.4774 (17)	0.4623 (17)	0.044 (6)*	
N5	0.32700 (18)	0.63601 (13)	0.41351 (11)	0.0365 (3)	
H5N	0.258 (3)	0.6232 (18)	0.3654 (17)	0.055 (6)*	
N6	0.43008 (17)	0.60804 (13)	0.58842 (11)	0.0407 (4)	
O1	0.16275 (15)	0.42397 (11)	0.71839 (9)	0.0458 (3)	
O2	0.06654 (16)	0.51991 (11)	0.86283 (9)	0.0481 (3)	
O3	−0.03729 (15)	0.57920 (10)	0.68927 (9)	0.0455 (3)	
O4	−0.11722 (15)	0.39661 (11)	0.75484 (9)	0.0470 (3)	

### Atomic displacement parameters (Å^2^)

**Table:**

	*U*^11^	*U*^22^	*U*^33^	*U*^12^	*U*^13^	*U*^23^
S	0.0389 (2)	0.0320 (2)	0.0269 (2)	−0.00002 (16)	−0.00122 (16)	0.00050 (15)
C1	0.0331 (8)	0.0321 (8)	0.0402 (9)	−0.0035 (6)	0.0027 (7)	0.0042 (7)
C2	0.0478 (10)	0.0425 (10)	0.0379 (10)	−0.0031 (8)	0.0115 (8)	0.0015 (7)
C3	0.0645 (12)	0.0507 (12)	0.0324 (9)	−0.0060 (9)	−0.0003 (8)	0.0056 (8)
C4	0.0527 (11)	0.0497 (11)	0.0454 (11)	−0.0019 (9)	−0.0103 (9)	0.0149 (8)
C5	0.0334 (8)	0.0348 (9)	0.0300 (8)	0.0057 (7)	0.0039 (6)	−0.0010 (6)
C6	0.0446 (10)	0.0397 (10)	0.0475 (10)	0.0027 (8)	0.0106 (8)	0.0083 (8)
C7	0.0492 (11)	0.0425 (11)	0.0611 (12)	−0.0085 (9)	0.0010 (9)	0.0039 (9)
C8	0.0470 (10)	0.0452 (11)	0.0496 (11)	−0.0040 (9)	−0.0064 (8)	−0.0052 (9)
N1	0.0529 (10)	0.0413 (9)	0.0402 (9)	0.0099 (7)	−0.0023 (7)	−0.0033 (7)
N2	0.0327 (7)	0.0390 (8)	0.0359 (8)	0.0016 (6)	0.0011 (6)	0.0042 (6)
N3	0.0454 (8)	0.0429 (9)	0.0468 (9)	0.0057 (7)	−0.0030 (7)	0.0083 (7)
N4	0.0437 (9)	0.0510 (10)	0.0318 (8)	−0.0089 (7)	0.0005 (7)	0.0033 (7)
N5	0.0367 (8)	0.0414 (8)	0.0306 (8)	0.0012 (6)	0.0013 (6)	0.0015 (6)
N6	0.0427 (8)	0.0446 (9)	0.0334 (7)	−0.0002 (6)	−0.0012 (6)	−0.0012 (6)
O1	0.0516 (7)	0.0509 (8)	0.0354 (7)	0.0103 (6)	0.0074 (5)	0.0021 (5)
O2	0.0541 (8)	0.0532 (8)	0.0334 (7)	0.0134 (6)	−0.0093 (5)	−0.0125 (5)
O3	0.0528 (7)	0.0358 (7)	0.0451 (7)	−0.0001 (5)	−0.0055 (6)	0.0084 (5)
O4	0.0518 (8)	0.0488 (8)	0.0387 (7)	−0.0128 (6)	−0.0012 (5)	0.0086 (5)

### Geometric parameters (Å, º)

**Table:**

S—O3	1.4656 (12)	C5—N6	1.350 (2)
S—O1	1.4675 (13)	C5—N5	1.352 (2)
S—O4	1.4709 (12)	C6—C7	1.343 (3)
S—O2	1.4710 (12)	C6—N5	1.347 (2)
C1—N1	1.316 (2)	C6—H6A	0.9300
C1—N3	1.347 (2)	C7—C8	1.392 (3)
C1—N2	1.356 (2)	C7—H7A	0.9300
C2—N2	1.343 (2)	C8—N6	1.314 (2)
C2—C3	1.353 (3)	C8—H8A	0.9300
C2—H2A	0.9300	N1—H1NA	0.86 (2)
C3—C4	1.390 (3)	N1—H1NB	0.91 (2)
C3—H3A	0.9300	N2—H2N	0.86 (2)
C4—N3	1.318 (2)	N4—H4NA	0.81 (2)
C4—H4A	0.9300	N4—H4NB	0.78 (2)
C5—N4	1.308 (2)	N5—H5N	0.80 (2)
			
O3—S—O1	110.49 (8)	C7—C6—H6A	120.2
O3—S—O4	108.64 (7)	N5—C6—H6A	120.2
O1—S—O4	109.60 (8)	C6—C7—C8	117.11 (17)
O3—S—O2	110.46 (7)	C6—C7—H7A	121.4
O1—S—O2	109.27 (7)	C8—C7—H7A	121.4
O4—S—O2	108.34 (8)	N6—C8—C7	124.08 (17)
N1—C1—N3	119.53 (16)	N6—C8—H8A	118.0
N1—C1—N2	119.43 (16)	C7—C8—H8A	118.0
N3—C1—N2	121.03 (16)	C1—N1—H1NA	118.1 (14)
N2—C2—C3	119.86 (17)	C1—N1—H1NB	119.0 (14)
N2—C2—H2A	120.1	H1NA—N1—H1NB	122.7 (19)
C3—C2—H2A	120.1	C2—N2—C1	121.12 (15)
C2—C3—C4	116.45 (18)	C2—N2—H2N	117.7 (13)
C2—C3—H3A	121.8	C1—N2—H2N	121.2 (13)
C4—C3—H3A	121.8	C4—N3—C1	116.88 (16)
N3—C4—C3	124.65 (16)	C5—N4—H4NA	118.6 (14)
N3—C4—H4A	117.7	C5—N4—H4NB	119.6 (15)
C3—C4—H4A	117.7	H4NA—N4—H4NB	122 (2)
N4—C5—N6	119.26 (15)	C6—N5—C5	121.10 (16)
N4—C5—N5	119.79 (15)	C6—N5—H5N	118.3 (15)
N6—C5—N5	120.95 (15)	C5—N5—H5N	120.4 (15)
C7—C6—N5	119.64 (17)	C8—N6—C5	117.11 (15)

### Hydrogen-bond geometry (Å, º)

**Table:**

*D*—H···*A*	*D*—H	H···*A*	*D*···*A*	*D*—H···*A*
N1—H1*NA*···O1	0.86 (2)	1.99 (2)	2.853 (2)	175.5 (17)
N1—H1*NB*···O3^i^	0.91 (2)	1.97 (2)	2.882 (2)	176.9 (2)
N2—H2*N*···O2	0.86 (2)	1.79 (2)	2.640 (2)	174.7 (18)
N4—H4*NA*···O1	0.81 (2)	2.10 (2)	2.902 (2)	167.6 (19)
N4—H4*NB*···O3^ii^	0.78 (2)	2.19 (2)	2.962 (2)	171.0 (2)
N5—H5*N*···O4^ii^	0.80 (2)	1.84 (2)	2.631 (2)	172.6 (2)
C2—H2*A*···O4^iii^	0.93	2.50	3.295 (2)	144
C3—H3*A*···N6^iv^	0.93	2.58	3.382 (2)	145
C4—H4*A*···O1^v^	0.93	2.57	3.231 (2)	128
C7—H7*A*···O2^vi^	0.93	2.51	3.101 (2)	121
C8—H8*A*···O4^vii^	0.93	2.59	3.237 (2)	127

Symmetry codes: (i) −*x*+1/2, *y*−1/2, −*z*+3/2; (ii) −*x*, −*y*+1, −*z*+1; (iii) −*x*, −*y*+1, −*z*+2; (iv) −*x*+1, −*y*+1, −*z*+2; (v) *x*+1/2, −*y*+1/2, *z*+1/2; (vi) *x*+1/2, −*y*+3/2, *z*−1/2; (vii) −*x*+1/2, *y*+1/2, −*z*+3/2.
